# Diabetes and Alzheimer's Disease

**DOI:** 10.1111/1753-0407.70103

**Published:** 2025-05-19

**Authors:** Zachary Bloomgarden

**Affiliations:** ^1^ Department of Medicine, Division of Endocrinology, Diabetes and Bone Disease Icahn School of Medicine at Mount Sinai New York New York USA

The relationship between diabetes and Alzheimer's Disease (AD) has increasingly been recognized. Diabetes is associated with the doubling of vascular dementia and with a one‐third increase in the risk of AD [1]. AD prevalence and mortality have particularly increased over the past three decades in China, and among women in relation to increases in longevity, with higher levels of glycemia the major attributable risk factor, with further risk associated with cigarette use and obesity [2], at least in part reflecting the association of all three of these factors with insulin resistance. During this time period, obesity has shown greater attributable risk while smoking has become a weaker risk factor, while other factors including environmental pollutants, nutritional deficiencies, alcohol use, and hypertension appear to be associated with considerably lower population attributable risk [2].

Insulin plays a variety of roles in neuronal function and survival, with diabetes increasing AD risk indirectly as a function of underlying brain insulin resistance, leading to impaired cognitive processes and increasing AD susceptibility [3]. In insulin‐resistant states, brain insulin levels rise, leading to reduced insulin‐degrading enzyme (IDE) activity. IDE is responsible for clearing Amyloid‐beta (Aβ). Aβ monomers play roles in neuronal synaptic activity, but Aβ has a tendency to autoaggregate, with reduction in IDE activity resulting in greater levels of Aβ, promoting plaque formation and contributing to AD pathology from Aβ accumulation, aggregation, and fibril formation [4]. Tau protein functions by stabilizing neuronal microtubules and plays a role in neuronal cell signaling. Insulin resistance downregulates an insulin signaling pathway, leading to decreased phosphoinositide 3‐kinase (PI3K) activity, in turn altering the activity of the serine/threonine kinase Akt pathway, leading to activation of glycogen synthase kinase‐3β (GSK‐3β). In addition to its function in regulating glycogen synthesis, GSK‐3β is an enzyme involved in phosphorylation of tau protein, with hyperphosphorylated tau aggregating to form neurofibrillary tangles [4]. The typical pathologic findings of AD, then, are exacerbated by insulin resistance, underlying the association of diabetes with AD.

Both among individuals having and not having diabetes, higher average glucose levels are associated with an increased hazard ratio for dementia [5]. Similarly, higher HbA1c levels are also associated with greater risk of dementia in patients with diabetes [6]. There may be relationships between diabetes treatment approaches and dementia development. Of concern, sulfonylureas were associated with a higher risk of dementia development than dipeptidyl peptidase 4 inhibitors (DPP4i) [7]. The glucagon‐like protein‐1 receptor agonists (GLP‐1 RAs) may reduce brain Aβ levels and tau hyperphosphorylation by activating Akt and mitochondrial Peroxisome proliferator‐activated receptor‐gamma coactivator‐1α, the latter suppressing oxidative stress and neuroinflammation, leading to decreased Aβ production [6]. A meta‐analysis of 23 population studies indicated that GLP‐1 RAs may reduce the risk of dementia or cognitive impairment [8]. Clinical trials are underway to investigate the potential of the GLP‐1 RA semaglutide in modifying AD among early‐stage symptomatic patients [9]. The sodium‐glucose co‐transporter 2 Inhibitors (SGLT2i) may also have neuroprotective antioxidant and anti‐inflammatory effects, increasing neurogenesis and synaptic activity and decreasing ischemic neuronal damage and mitochondrial dysfunction, as well as improving hyperglycemia and insulin sensitivity [10]. Several studies have indicated that SGLT2 inhibitors are associated with a reduced risk of dementia compared to dipeptidyl peptidase 4 (DPP4) inhibitors [11, 12], the latter study showing specific evidence of reduction in AD as well as in vascular dementia development, with a meta‐analysis of clinical studies further supporting this potential benefit [8]. In a recent population study among some 34 000 people with diabetes, use of GLP‐1RA and SGLT2i were associated with 33% and 43% lower likelihood of dementia development, respectively [13]. Pioglitazone is an insulin sensitizer which also might be thought to improve factors involved in AD pathogenesis, although limited clinical trials have not shown reduction in dementia [6]. Metformin has been shown to improve insulin resistance and may also affect tau phosphorylation through the mammalian target of rapamycin (mTOR)/Protein Phosphatase 2A (PP2A) pathway [6]. Vitamin D deficiency has neurological effects, potentially contributing to AD [14], with evidence of an association between vitamin D supplement use and reduction in dementia development [15].

There is, then, a strong connection between diabetes and AD, reflecting underlying insulin resistance leading to Aβ accumulation and tau hyperphosphorylation. Appropriately powered clinical trials of GLP‐1 RAs and SGLT2 inhibitors, as well as of further potential therapies, are needed to determine effective strategies for prevention and treatment. Conceptually, physical activity and a healthy diet can improve insulin sensitivity and should be effective in reducing AD, but existing evidence to develop effective lifestyle approaches is limited [16, 17, 18], and this too appears to be an important potential area for research.

## Conflicts of Interest

The author declares no conflicts of interest.
